# Voltage readout from a piezoelectric intracochlear acoustic transducer implanted in a living guinea pig

**DOI:** 10.1038/s41598-019-39303-1

**Published:** 2019-03-06

**Authors:** Chuming Zhao, Katherine E. Knisely, Deborah J. Colesa, Bryan E. Pfingst, Yehoash Raphael, Karl Grosh

**Affiliations:** 1 Department of Mechanical Engineering, University of Michigan, Ann Arbor, MI 48109 USA; 2 Kresge Hearing Research Institute, Department of Otolaryngology - Head and Neck Surgery, University of Michigan, Ann Arbor, MI 48109 USA; 3 Department of Biomedical Engineering, University of Michigan, Ann Arbor, MI 48109 USA

## Abstract

The ability to measure the voltage readout from a sensor implanted inside the living cochlea enables continuous monitoring of intracochlear acoustic pressure locally, which could improve cochlear implants. We developed a piezoelectric intracochlear acoustic transducer (PIAT) designed to sense the acoustic pressure while fully implanted inside a living guinea pig cochlea. The PIAT, fabricated using micro-electro-mechanical systems (MEMS) techniques, consisted of an array of four piezoelectric cantilevers with varying lengths to enhance sensitivity across a wide frequency bandwidth. Prior to implantation, benchtop tests were conducted to characterize the device performance in air and in water. When implanted in the cochlea of an anesthetized guinea pig, the *in vivo* voltage response from the PIAT was measured in response to 80–95 dB sound pressure level 1–14 kHz sinusoidal acoustic excitation at the entrance of the guinea pig’s ear canal. All sensed signals were above the noise floor and unaffected by crosstalk from the cochlear microphonic or external electrical interference. These results demonstrate that external acoustic stimulus can be sensed via the piezoelectric voltage response of the implanted MEMS transducer inside the living cochlea, providing key steps towards developing intracochlear acoustic sensors to replace external or subcutaneous microphones for auditory prosthetics.

## Introduction

According to estimates from the World Health Organization, 360 million people in the world had impaired hearing, as of 2011^1^. Cochlear implants (CIs) are an effective therapeutic solution for treating sensorineural hearing loss. Most commercially available CIs have an external microphone/processing unit, a radio frequency (RF) inductive link, and intracochlear electrodes. Although the CIs enable speech recognition, they have major limitations including high cost, high power consumption (20–40 mW), cosmetic concerns, and safety issues associated with the external processing unit^2–4^. These limitations contribute to an international market penetration for CIs of approximately 0.7%^5^. The development of a fully implantable cochlear implant is attractive, because it would improve cosmetic and safety issues. Unlike the traditional CI, where external components must be removed for many activities including sleep and showering^6^, a fully implantable CI could remain on at all times. Additionally, a fully implantable cochlear implant would eliminate the inductive link between the processor and CI electrodes that causes 60% of the power drop^2^ in the device, thus reducing power consumption^4^.

There have been several attempts to build fully implantable cochlear implants^7–12^. Each requires an internal microphone of some kind. For instance, Cochlear Corporation developed the Totally Implantable Cochlear Implant (TIKI), which used a subcutaneous microphone. In a clinical study of this device^10^, subjects reported benefits from TIKI and had continued to use it on a daily basis because of cosmetic advantages and the ability to hear while showering, sleeping, and doing physical work. However, speech perception results were significantly lower when compared with the traditional CI because of the reduced sensitivity and increased body noise contamination of the subcutaneous microphone. The state of the art for implantable acoustic sensors for hearing devices is well-reviewed in^13^ where they also present a trans-tympanic microphone to measure ear canal pressure for hearing aids or cochlear implants. This approach, while promising, also faces issues such as ventilation tube migration and liquid contamination which would reduce the effectiveness of the device^13^. An intracochlear transducer is an attractive alternative for patients with a functioning middle ear, because the intracochlear acoustic pressure is typically higher than the ear canal pressure^14–17^. Olson^14^, using a fiber-optic probe with a polymer-gold membrane, and Dancer and Franke^17^, using a Kulite piezoresistive sensor coupled to a small silicone-filled probe tube, successfully measured the fluctuating pressures inside a living cochlea. These measurements serve as a reference for the pressures measured in the current study, although neither of these systems is robust or small enough for chronic use. Creighton *et al*.^18^ and Pfiffner *et al*.^19^ implanted a miniature microphone in the cadaveric human temporal bone and measured intracochlear sound pressure *in vitro*. Recently a PVDF-based intracochlear microphone prototype was developed and used to measure sound in a living gerbil cochlea^20^. In the current study, we present a different approach to fabricating an implantable acoustic transducer, one based on an aluminum nitride (AlN) micro-electro-mechanical systems (MEMS) xylophone along with physiological testing in a living guinea pig to sense the voltage response due to external acoustic excitation.

In part, our efforts to build the piezoelectric intracochlear acoustic transducer (PIAT) were informed by research on piezoelectric artificial basilar membranes (ABMs). Recent development of microfabrication techniques enabled the development of the life scale ABM models^21–24^ with tonotopicity (place-to-frequency mapping). Shintaku *et al*.^25^ fabricated a piezoelectric ABM that had a frequency selective electrical response to acoustic stimuli. This transducer was then used as the *ex vivo* (external) front end of a cochlear implant to evoke auditory brain-stem responses^26^. In the same paper^26^, these investigators fitted a piezoelectric transducer into an excised cochlea and measured the plate vibration in response to stapes excitation using an external laser vibrometer. Such an approach could not be applied to an *in vivo* experiment due to the use of the laser vibrometer and the large fenestra required for inserting the piezoelectric plate. A different approach to making an artificial cochlea is the development of xylophone-like structures with arrays of cantilevers which resonate at different frequencies^27–29^. Jang, *et al*.^30,31^ coupled an *ex vivo* piezoelectric cantilever array transducer to signal processing hardware which generated biphasic current pulses. The output was connected to a deafened animal via a cochlear implant electrode array to successfully evoke auditory brainstem responses.

Recently, very small low noise piezoelectric microphones using MEMS fabrication techniques have been developed by Littrell and Grosh^32,33^. Here, we report on an extension of this in-air work by creating a PIAT designed to sense sound inside the cochlea of a guinea pig *in vivo* caused by an external acoustic excitation. Unlike some previous piezoelectric MEMS sensors used for hearing applications^25,26,30,31^, our design was scaled to enable *in vivo* implantation in a guinea pig for acute studies of the effectiveness of the device. The piezoelectric material AlN was chosen to sense the acoustic pressure because of its low dielectric loss, compatibility with MEMS fabrication, and biocompatibility for chronic *in vivo* study^32,34,35^. Some preliminary design, fabrication and benchtop testing results of the transducer were reported previously^36–39^. In the work reported here, we further applied waterproof coating to the transducer and characterized its effect on the PIAT confirming the functionality in air and underwater. The PIAT was then implanted in the cochlea of a living guinea pig, and electrical signals from the PIAT were sensed in response to external acoustic excitation. *Post mortem* analysis was conducted to examine the PIAT condition after the *in vivo* tests. Remarkably, the excised PIAT was still partially functional when retrieved from the cochlea.

## Results

### Fabrication of AlN Cantilever Array for the PIAT

**Figure 1:**
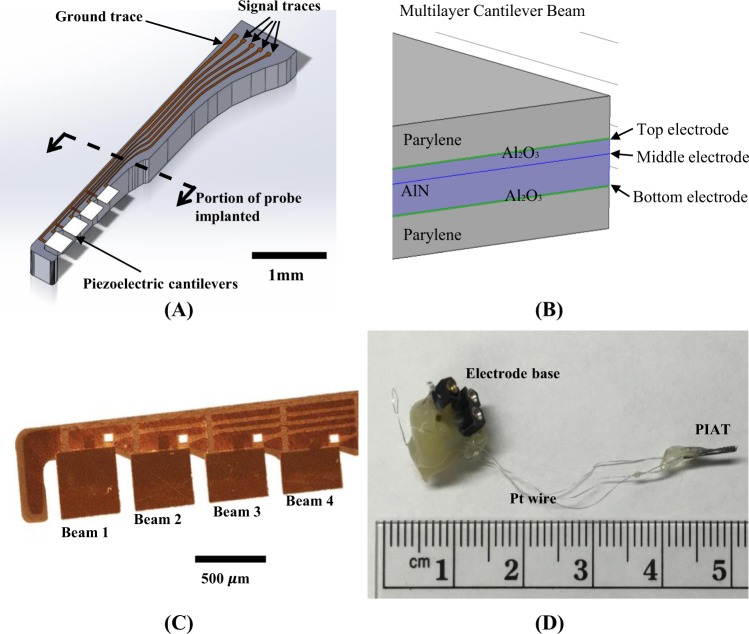
The designed and fabricated PIAT. (**A**) A 3D drawing of the PIAT, which consists of four piezoelectric cantilevers with variable lengths on a silicon shank. (**B**) A cross-sectional view of the multilayer cantilever beam. (**C**) A micrograph of the implantable portion of a fabricated probe. (**D**) A fabricated PIAT bonded with Pt wires and a percutaneous connector.

The PIAT probes were fabricated^38,39^ using the process described in the Methods section as summarized in Fig. S1. Briefly, the PIAT probe consists of four 400 *μ*m wide piezoelectric cantilevers of different lengths that span 300 *μ*m to 443 *μ*m, as given in Table 1, in order to achieve fluid-loaded resonance frequencies over a range of 5.6–13.5 kHz. A 3D rendering of the design of the PIAT is pictured in Fig. 1A. Once the probes were released from the wafer, they were coated with 50 nm atomic layer deposition (ALD) alumina and 2 *μ*m parylene to enhance the durability and waterproofing of the device. In Fig. 1B the cross section of the multilayer AlN cantilever beam is shown. A close-up photograph of the sensing portion of the released probe is shown in Fig. 1C. The entire device is shown in Fig. 1D including the Pt-Ir wires as well as the silicone insulation of electrical connections at the percutaneous connector and the shank of the PIAT. The signal and ground traces of the PIAT probes were bonded to Pt-Ir wires as described in the Methods section.

**Table 1:** Beam length, resonant frequencies (*f*_r_), and quality factors (*Q)* in air and in water after the alumina/parylene coating.

	Beam Length (*µ*m)	In air		In water	
		*f*_*r*_ (kHz)	*Q*	*f*_*r*_ (kHz)	*Q*
Beam 1	443	18.8	90	5.6	3.5
Beam 2	397	23.8	47	7.1	3.9
Beam 3	345	31.6	53	9.9	4.4
Beam 4	300	40.8	56	13.5	4.7

Before performing more complete actuation and sensing tests of the device, some fundamental response metrics were characterized. Using previously reported techniques^32,33^, the piezoelectric coupling coefficient *d*_31_ of the AlN was determined to be −1.0 pm/V. This is within the range measured by others (−0.9 to −2.8 pm/V)^33,40–42^. We tested the electrical insulation of the PIAT by measuring the electrical impedance of the PIAT using an impedance analyzer (Agilent E4980A) at 1 kHz both in air and during submersion in saline. The measured electrical impedance was the same under both conditions (a capacitance of *C*_*d*_ = 38 pF and a parallel resistance of *R*_*p*_ = 81 MΩ). This impedance remained unchanged through a 5 hour submersion in saline, demonstrating the effectiveness of the coating.

### Benchtop Actuation Testing

**Figure 2:**
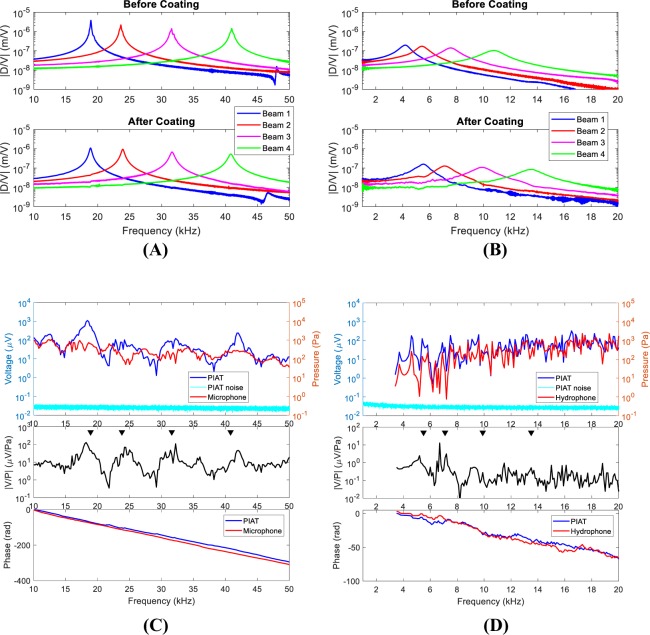
Benchtop actuation and sensing tests. (**A**) Actuation test in air. The beam tip displacement in air in response to voltage actuation before (upper) and after (lower) alumina/parylene coating. (|D/V| represents the ratio of displacement to voltage amplitude). (**B**) Actuation test in water. The beam tip displacement in water in response to voltage actuation before (upper) and after (lower) alumina/parylene coating. (**C**) Sensing test in air. The upper panel shows the voltage output from the PIAT (blue line with units on the left axis), the device noise floor (cyan line with units on the left axis), and the acoustic pressure measured by microphone (red line with units on the right axis). The middle panel shows the transfer function of the measured voltage from the PIAT and the measured pressure from the microphone. |V/P| is the amplitude of voltage to pressure ratio. The resonances of the beams in air from actuation tests are shown as black triangles for comparison. The phase plot in the lower panel shows the phase difference between the speaker-driving voltage and the measured signal by the PIAT (blue line) or microphone (red line). (**D**) Sensing test in water. The upper panel shows the voltage output from the PIAT (blue line with units on the left axis), the device noise floor (cyan line with units on the left axis), and the acoustic pressure measured by hydrophone (red line with units on the right axis). The middle panel shows the transfer function of the measured voltage from the PIAT and the measured pressure from the hydrophone. The resonances of the beams in water from actuation tests are shown as black triangles for comparison. The phase plot in the lower panel shows the phase difference between the speaker driving voltage and the measured signal by the PIAT (blue line) or hydrophone (red line).

In order to verify the device functionality, we first used the converse piezoelectric effect, imposing a voltage across the PIAT electrodes and measuring the vibration response. In Fig. 2A, the in-air frequency response of the displacement of the tip of each beam normalized to the input voltage is plotted, showing clear peaks at each beam resonance. Before the parylene/alumina coating was applied (upper panel in Fig. 2A), the in-air beam resonant frequencies of the four beams were 18.9, 23.7, 31.5, and 41.0 kHz in air with maximum tip displacement to voltage ratios ranging from 1–4 *μ*m/V at resonance. The frequency response of the beams coated with ALD alumina and parylene C is shown in the lower panel. After the coating, the peak displacements were reduced to 0.4–1 *μ*m and the resonant frequencies shifted about 2%. This small frequency change was due to the balancing of the added mass-loading effect of the compliant parylene coating and the stiffening caused by the ALD alumina coating. The stiffening effect was mainly due to the residual stress of the ALD deposition^43–46^.

The frequency responses of the electrically excited beams submerged in water are shown in Fig. 2B. The measured frequency response without the alumina/parylene coating is shown in the upper panel and the fluid-loaded frequency response is shown in the lower panel. The measured resonance frequency (*f*_*r*_) and quality factor *Q* (where $Q=\frac{{f}_{r}}{{\rm{\Delta }}f}$ and $\triangle f$ is the full width at half maximum) of the cantilevers in air and water are summarized in Table 1. The fluid-loaded resonance frequencies were reduced by approximately 65–75% of the in-air values, and the *Q* was decreased significantly. The largest peak displacement at resonance was reduced to 0.2 *μ*m underwater. The low frequency displacement of each beam, however, was unchanged from the in-air value. After the alumina/parylene coating was applied, as shown in the lower panel in Fig. 2B, the resonances increased as much as 31% in water compared to uncoated values. The reason for this increase is that when the PIAT was submerged in water, the mass-loading of the water dominated that of the parylene coating. Therefore, there was little change to the mass-loading of the uncoated versus coated fluid-loaded beam resulting in an increased resonant frequency in water due to the ALD stiffening.

### Benchtop Sensing Testing

Next, the voltage response of the PIAT to acoustic excitation was measured both in air and in water. The tests were conducted as described in the Methods. In the upper panel of Fig. 2C the piezoelectric voltage output from the PIAT in response to in-air sound stimulus (blue line) and the device noise floor (cyan line) are shown using the left-hand-side axis for the ordinate. The measured voltage was clearly above the noise floor with a signal to noise ratio (SNR) of at least 35 dB. The acoustic pressure measured by a microphone (red line) is shown using the right-hand-side axis for the ordinate. The transfer function between the measured voltage output from the PIAT and the acoustic pressure from the microphone is shown in the middle panel of Fig. 2C. In this middle panel, the frequencies of the amplitude peaks matched well with the voltage actuated resonances (shown as black triangles). The voltage minima between the resonance frequencies were due to the out-of-phase summation of currents generated by adjacent beams. This is because the longer, lower-frequency beam will respond out of phase with the pressure excitation for frequencies above its resonance while the neighboring, shorter beam will respond in phase with the pressure (below its first resonant frequency). The lower panel shows the phase response of the sensor (for either the PIAT or the microphone) referred to the drive voltage sent to the speaker (note that this is not the phase of the sensitivity). This phase was dominated by the acoustic delay from the speaker to the sensor. We used the slope of the phase to determine a delay of 1.14 ± 0.002 ms as measured by the PIAT and 1.19 ± 0.003 ms as measured by microphone. These delays corresponded to distances of 0.39 ± 0.0007 m and 0.41 ± 0.001 m for sound travelling in air as measured by microphone and the PIAT, respectively. Because the delay of the PIAT output corresponded to the appropriate acoustic delay and the resonant amplitudes match the driven resonant frequencies of the probe, we are confident that the response was not contaminated with cross talk from the drive signal to the loudspeaker. This test demonstrated the sensing functionality of the PIAT in air.

Sensing in water was tested for frequencies ranging from 3.5–20 kHz with the results shown in Fig. 2D. Similar to Fig. 2C, the upper panel shows the voltage output from a PIAT (blue line) and the device noise floor (cyan line) both referred to the axis on the left, as well as the acoustic pressure measured by a hydrophone (red line) referred to the axis on the right. The measured voltage was at least 15 times higher than the noise floor (corresponding to an SNR of at least 23 dB). Unlike sensing in air, both the voltage response and pressure response show many fluctuations through the measured frequency range because of the modal nature of the water tank. The low frequency, fine-structure peaks in the voltage response (blue) align well with the peaks in the acoustic pressure response (red) as the hydrophone was placed at nearly the same location as the PIAT; the correlation begins to degrade at high frequencies because of slight placement errors. The middle panel shows the transfer function of the measured voltage from the PIAT and the measured acoustic pressure from hydrophone. Compared to sensing in air in Fig. 2C, the transfer function amplitude peaks due to beam resonances were less prominent because of the damping of the fluid (as seen in Fig. 2B) and the confounding effect of the water tank resonances (as seen in the upper panel in Fig. 2D). However, the peaks around 5.6 kHz and 7.1 kHz were observed, which corresponded to the fluid-loaded resonances of Beams 1 and 2 measured under direct electrical stimulation (the voltage-actuated resonances are shown as black triangles). The peaks corresponding to the resonances of Beams 3 and 4 were less pronounced, probably because the beams were located closer to the pressure release, air-water interface where acoustic pressure was smaller. We confirmed this decrease in pressure by moving the probe 1 cm closer to the water surface and found that the PIAT voltage output was reduced by 50%. The sensor phase relative to the excitation voltage for the underwater source (for the PIAT or hydrophone) is shown in the lower panel. In the 8–20 kHz range, a delay of 0.62 ± 0.03 ms was measured by the PIAT and a 0.64 ± 0.02 ms delay was measured by the hydrophone. The difference in the delays was within the slope uncertainty of our estimate of the delay. This test confirmed the sensing functionality of the PIAT when directly exposed to a water environment.

### *In Vivo* Response

The PIAT was implanted in the cochlea of an anesthetized guinea pig, as described in the Methods. After the implantation surgery, the transducer impedance was measured and compared to the impedance measured before the surgery in order to check the electrical integrity of the device. A parasitic parallel capacitance *C*_*p*_ = 3 pF was measured in addition to the device capacitance *C*_*d*_ = 38 pF. The parasitic parallel resistance *R*_*p*_ decreased from 81 MΩ to 10 MΩ at 1 kHz. This change in impedance decreased the device sensitivity by no more than 10%. These results indicate the PIAT was successfully implanted with some minor compromise to the device performance.

**Figure 3:**
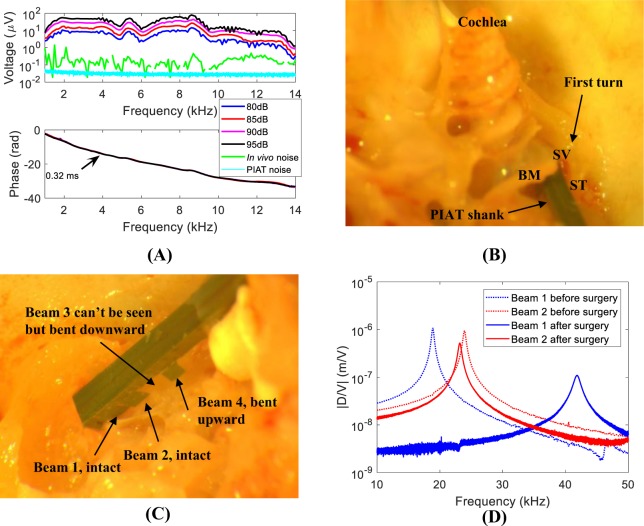
*In vivo* testing results. (**A**) The *in vivo* frequency response with different SPL inputs, *in vivo* noise and intrinsic PIAT noise floor measured in a closed sound attenuating chamber. A 0.32 ms delay, derived from the phase slope, agrees with the acoustic delay from the speaker to the PIAT. (**B**) A picture from the post mortem dissection showing the location of the PIAT probe placed inside the guinea pig cochlea. (**C**) A picture showing the four beams after removal from the cochlea. (**D**) Comparison of in-air actuation transfer functions for Beams 1 and 2 before implantation surgery and after removal from the cochlea. (|D/V| is the ratio of the amplitude of the displacement to input voltage).

After confirming the device was properly implanted and functioning, the *in vivo* voltage response of the PIAT to pure tone acoustic stimuli was measured, as shown in Fig. 3A. The external acoustic excitation spanned from 1–14 kHz and ranged from 80–95 dB sound pressure level (SPL) with a 5 dB difference of each step. The voltage output of the PIAT in response to the this input was linear because the sensor itself is linear and the active processes responsible for cochlear nonlinearity were rendered inoperable or certainly made less efficient by the invasive nature of this surgery^47^. In addition, the input-output relations of the cochlea to a pure tone at relatively high SPL are also typically nearly linear^47^. Voltage outputs of 1.3–79.7 *μ*V from the PIAT with an SNR that varied from 17 dB to 70 dB were measured in response to a 95 dB SPL input stimuli over frequencies of 1–14 kHz. The PIAT showed a higher response around 2–9 kHz, near to the fluid-loaded resonances of Beams 1 and 2. The phase showed an accumulation consistent with the delay associated with the travel time from the speaker to the cochlea, as shown in the lower panel of Fig. 3A, which indicated that the signal collected was not noise or crosstalk (e.g., crosstalk would typically have a constant phase value). The voltages of 1.3–79.7 *μ*V in response to the 95 dB SPL input (or 1.2–71.0 *μ*V/Pa) measured *in vivo* in the cochlea were much higher than 0.05–12.6 *μ*V/Pa as measured in the benchtop testing outside the cochlea in a water tank to an excitation with the same acoustic pressure level (The *in vivo* results were compared to sensing in water because the scala tympani was filled with water-like perilymph). This observation is in line with the previous *in vivo* intracochlear pressure measurements in rodents (0–38 dB higher inside the scala tympani of a guinea pig^17^ and 10–50 dB higher for a gerbil^15,16^ as compared to the ear canal pressure).

The measured *in vivo* voltages were also compared to the noise levels of the sensor system and experimental test configuration. The *in vivo* noise was measured by recording the lock-in amplifier output from the implanted PIAT with no input to the speaker. The *in vivo* noise was due to the combination of electric noise of the testing system, intrinsic device noise, the cochlear ambient environment, and the amplifier noise. As seen in Fig. 3A, the *in vivo* noise level was below the sensed signal (~0.2 *μ*V in the 2–10 kHz range, and around 0.3 *μ*V in the 10–14 kHz range, with 80 dB SPL input). The intrinsic PIAT noise floor was measured in a sound isolation chamber prior to the surgery. The intrinsic device noise floor was even smaller than *in vivo* noise and was 0.03–0.04 *μ*V in the 1–14 kHz range as shown in Fig. 3A. Overall, all measured voltages were clearly greater than system noise.

After the *in vivo* sensing tests, the guinea pig was sacrificed and its cochlea was dissected to examine the condition of the PIAT. In Fig. 3B, the location of the implanted probe during the dissection is shown. In Fig. 3C, a close-up photograph showing the condition of the 4 cantilevers immediately after the extraction is presented. Beams 1 and 2 were intact while Beams 3 and 4 were significantly bent. Beam 4 was also buried in the muscle plug used to seal the cochleostomy. Under closer examination of Beam 1, we found it was slightly bent and partially covered with tissue at the base of this cantilever. In Fig. 3D, the tip displacement of the excised probe in response to voltage excitation (as in Fig. 2A) is presented and compared to the results prior to implantation. *Ex vivo* actuation responses indicated that Beam 2 was still fully functional, while the resonant frequency of Beam 1 was shifted to a higher frequency (possibly due to stiffening from the attached tissue). Beams 3 and 4 were still attached but not functional. The *ex vivo* test and *post mortem* analysis revealed that Beam 1 and 2 were certainly operational during the entire *in vivo* test. This is in line with the elevated measured voltage response near the resonances of Beams 1 and 2. It is, however, unclear when Beams 3 and 4 were damaged.

We considered the possibility that the cochlear microphonic (CM) could contaminate the measured *in vivo* voltage. Fridberger *et al*.^48^ found that the electrical potential in the organ of Corti in the base of a very healthy animal could be as high as 1 mV (7–22 kHz) in response to an 80 dB input stimulus. Our CM levels should be lower because of the damage to the cochlea from the implant surgery (as shown in the Hearing Condition for the Tested Animals in the Methods). To test for CM contamination we performed separate experiments. We measured the response of the PIAT to a pure tone sinusoidal signal of 1 mV (1–10 kHz) applied to a submerged electrode in a saline-filled dish 5 cm away from the PIAT. The output from the PIAT was recorded using the same lock-in amplifier as in the *in vivo* experiments. The largest signal detected due to this contamination signal was 0.3 *μ*V, which was near the *in vivo* noise level (~0.2 *μ*V in the 1–10 kHz frequency range), but below the sensed signals (2.8–79.7 *μ*V in the 1–10 kHz frequency range). Therefore, we estimate that the *in vivo* sensed signal was unaffected by the CM.

## Discussion

We have shown that a fully implantable PIAT that has the capability to read out the voltage in a living cochlea in response to external acoustic excitation can be fabricated using MEMS technology. Because the intracochlear pressure signals are expected to be higher than those outside the ear^15–17^ and certainly higher than those reaching a subcutaneous microphone, the PIAT has the potential to improve a fully implantable cochlear implant. We found that the transfer function between the applied ear canal acoustic pressure and the voltage output from the PIAT measured in the living cochlea fell in the range 1.2–71.0 *μ*V/Pa (computed from results presented in Fig. 3D) while the PIAT voltage to pressure ratio measured during the benchtop experiment ranged from 0.05–12.6 *μ*V/Pa (from Fig. 2D). This result indicates that the acoustic pressure inside the cochlea is higher than outside the cochlea. This result is in line with previous measurements^14–17^. All recorded *in vivo* voltage responses were above the noise and unaffected by the crosstalk from the loudspeaker, electrical interference, or the cochlear microphonic. The *ex vivo* actuation test confirmed that the PIAT probe was still partially functional after the *in vivo* testing. This indicates the robustness of the PIAT in spite of the potentially destructive surgeries. Taken as a whole, we successfully measured voltage readout with the PIAT implanted in the living cochlea and have provided important steps (for both manufacture and implantation) towards utilizing the intracochlear acoustic signal for future fully-implantable CIs.

Potential uses for the technology described in this paper include a sensor inside the cochlea. For patients with functioning middle ears, a PIAT could eliminate the need for an external microphone in a traditional CI. An internal sensor would enhance the ease of use and improve the appearance. Because of these potential advantages, such an approach has been proposed and pursued by other groups (e.g., Inaoka *et al*.^26^, Jang *et al*.^31^, and Mukherjee *et al*.^49^). Another prosthetic application of this piezoelectric technology includes its use in reverse as a receiver/actuator (intracochlear speaker) for a hearing aid or in a hybrid electro-acoustic stimulator for a cochlear implant. Presently, the stimulation is extracochlear, either outside the tympanic membrane or in the middle ear^50,51^. The piezoelectric bimorph technology developed here (modified to improve its radiation efficiency) could be placed inside the cochlea to generate sound. Luo *et al*.^52,53^ demonstrated an intracochlear PZT actuator that evoked an auditory brainstem response by generating acoustic signals inside a cochlea, an exciting proof-of-concept for this approach. Compared to Luo *et al*.’s design, the PIAT holds advantages because it is lead-free, smaller, and produces a larger displacement. The PIAT can also be used either as a sensor or as a generator of sound for basic science studies such as in cochlear mechanics measurements^15^. In actuator mode, these devices could be used to produce a well-defined, focal disturbance inside the cochlea. Such a controlled source, in combination with cochlear mechanics response measurement techniques (e.g., laser vibrometry, optical coherence tomography^54,55^ or otoacoustic measurements^56^) could lead to a better understanding of the genesis of different types of otoacoustic emissions and their relation to cochlear function. By attaching a proof mass to the end of the piezoelectric bimorph, it can also be designed for use as a accelerometer for middle ear motion sensing^57,58^. Other potential applications of this technology include sensing and actuation for many biomedical applications such as cardiovascular and urologic diagnostic procedures, surgical procedures and monitoring of invasive treatments. Moreover, the multi-resonant characteristics could be taken advantage of for use as a microphone or hydrophone, or even a broadband sound silencer^59,60^.

Improvements for future generations of the PIAT are possible. The rigid straight silicon backbone, which prevents deeper insertion of the PIAT into the cochlea, could be made smaller and flexible. With a MEMS based flexible cable connection^39^, the electrical performance could also be improved by allowing for easier access to individual channels and reducing electrical parasitics by decreasing the distance of the MEMS sensor to the amplifier. Co-design of the amplifier circuit with the MEMS sensor will also decrease the minimum detectable signal improving function. Optimization of the MEMS sensor itself, (e.g., acoustically shielding the backside of the cantilevers) could improve sensitivity significantly, especially in a fluid environment^61^.

## Methods

### Fabrication Process of the PIAT

The probes were fabricated in the following manner, as summarized in Fig. S1. A 102 mm diameter p-type (100) Si wafer substrate of 500 *μ*m thickness was coated with 1 *μ*m of wet thermal oxide. The transducers were comprised of a five-layer stack, consisting of two 1.5 *μ*m of AlN layers laminated between three 15/30 nm Ti/Pt electrode layers. Alternating layers of metal layers and AlN layers were deposited. The Ti/Pt layers were sputtered (Lab 18–2, Kurt J. Lesker Company, USA), and patterned by photolithography using SPR 220 photoresist and CD 30 developer. The traces in the metal layers were 20 *μ*m wide in the implantable part (first 4 mm from the tip) and were widened to 40 *μ*m wide in the base, away from the tip. The AlN layers were deposited using a dual cathode S-Gun magnetron AlN sputter tool (AMS 2004, Tegal OEM group, USA) with bulk film stress targeted to 0 ± 150 MPa (Fig. S1A). The active area of the piezoelectric bimorph was limited to 45% of the distance from the root toward the tip of the cantilever to improve the input referred noise of the devices (adding more electrode area adds electronic noise in greater proportion than the increase in output signal^32,62^). The AlN cantilevers metal contacts were opened using an etch process that was a combination of Cl-based RIE (9400, LAM Research Corporation, USA) and heated (50 °C) etchant (AZ 400 K, Clariant, USA) etching, and the cantilever geometry was defined using Cl-based RIE etching.

The top layer of AlN was unintentionally overetched by 0.8 *μ*m (leaving 0.7 *μ*m on this layer) and a bimorph thickness of 2.2 *μ*m. An asymmetrical AlN bimorph was created and the top surfaces were roughened (Fig. S1B). We found that the lowest noise sensor was created by using the non-roughened bottom layer and using roughened top layers as a structural layer only. The top and middle layer were connected and served as ground layer, and the bottom layer was connected to the signal trace. A layer of 20/400 nm of Cr/Au was sputtered (Lab 18–2, Kurt J. Lesker Company, USA) and formed the electrode pads (100 *μ*m wide and 300 *μ*m long) for bonding the Pt-Ir wire (Fig. S1C). The probe backbone was then defined using a through-wafer DRIE (STS Pegasus 4, SPTS Technologies Ltd., USA), followed by a BHF oxide etch to release the cantilevers. Details of the fabrication can be found in Reference^36,38,39^. Two 50 *μ*m thick Pt-Ir wires with PFA insulation (776000, A-M system, USA) were bonded on the electrode pads. One wire was bonded on the ground trace and the other one was bonded on the pads where all signal traces were connected in parallel. The bonding utilized conductive epoxy (H20E-FC, Epoxy Technology, USA) and was followed by Silicone sealing (3140 RTV Silicone, Dow Corning, USA). After the MEMS fabrication, the probe was coated with a combination of 50 nm of thermal atomic layer deposition (ALD) alumina (Oxford OpAL ALD, Oxford Instrument, UK) and 2 *μ*m of parylene (PDS 2035CR, Special Coating System, USA) to enhance the long term reliability of the PIAT probe in an ionic fluid environment^63,64^ (Fig. S1D). All probes were fabricated in the Lurie Nanomanufacturing Facility at the University of Michigan.

### In Air and Underwater Actuation and Sensing Benchtop Testing

**Figure 4:**
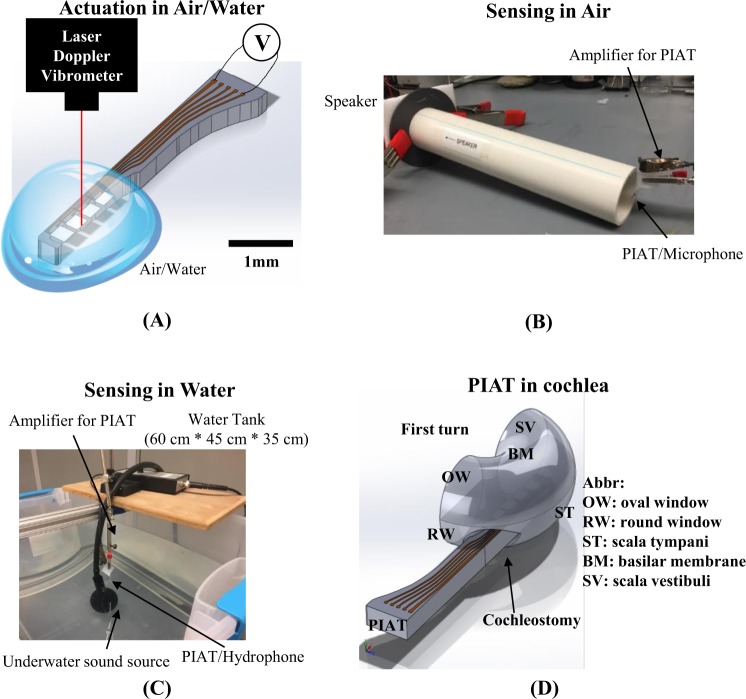
Schematics of test configurations. (**A**) Benchtop actuation test setup. Voltage was used to actuate each of the cantilever beams in air and water. The deflection at the tip of each beam was measured using a laser Doppler vibrometer (LDV). (**B**) Benchtop sensing test setup in air. Acoustic signals were generated by a piezo tweeter in air and passed through a 304.8 mm long PVC tube. The PIAT was placed at the end of the tube and the output was compared to that of a calibrated microphone. (**C**) Benchtop sensing test setup in air. Acoustic signals were generated by an underwater sound source in a water tank filled with tap water. The PIAT was placed near the water surface to sense the sound and compared with a calibrated hydrophone. (**D**) A CAD drawing illustrating the position of the PIAT as it passes through a cochleostomy into the scala tympani of a living cochlea.

The PIAT was tested in air and in water. Figure 4A showed the actuation test setup. The actuation tests were performed using LabVIEW 2009 controlled NI PCI-6251 card to apply voltage directly to the probe. The resulting tip deflections were measured using a laser Doppler vibrometer (LDV; OFV-303, Polytec, USA), with spot size 30 *μ*m. The device was mounted on a probe station (LA-150 DC, Semiprobe, USA) with a 2D micropositioning stage. The water actuation test was done with a drop of water delivered to the implantable portion of the PIAT probe, covering all cantilevers. A goniometer (123–2890, Optosigma, France) was used to adjust the angle of each cantilever beam for the best reflectivity. NI PCI-6123 card was used to measure the LDV response.

The sensing tests were done by applying acoustic excitation and measuring the voltage from the PIAT with a lock-in amplifier (SR830, Stanford Research System, USA), as illustrated in Fig. 4B,C. A custom-made amplifier (an instrumentation amplifier (MAX4462, Maxim Integrated, USA) and an operational amplifier (LT6233, Texas Instrument, USA)) were used to buffer and amplify the voltage signal. The amplifier gain of 100 was factored out of all the results presented in this paper. Figure 4B shows the setup for the in-air sensing test. A 12 inch (304.8 mm) long plastic tube was placed in between the PIAT and a piezo speaker (15D841, Motorola, USA). Pure tone sinusoidal signals were sent into the speaker and swept through 40–80 kHz. A calibrated microphone (2520, Larson Davis, USA) was used at the same location to measure the sound pressure level. As shown in Fig. 4C, underwater sensing tests were done by immersing the PIAT probe below the water surface of a tank (dimensions given in Fig. 4C) with approximately a 0.1 m^3^ volume of tap water and playing sounds (1–20 kHz) with an underwater transmitter (ITC-1032, Channel Technologies Group, USA). A hydrophone (TC4013, Teledyne Reson, USA) was used to measure the sound pressure level at the same location. The tap water was connected to the ground to eliminate electrical interference.

### Experimental Animal

The animal used in these acute experiments was an adult male specific pathogen free (SPF) pigmented guinea pig (600 g) bred and maintained by the Unit for Laboratory Animal Medicine at the University of Michigan. The animal use protocol was reviewed and approved by the University of Michigan Institutional Animal Care and Use Committee. All experiments were performed in accordance with relevant guidelines and regulations. The PIAT was implanted in the right ear. After all testing was complete, the animal was euthanized and the cochlea was dissected for confirmation of implant location and integrity as detailed below.

### Implantation Procedures

The animal was anesthetized with ketamine (40 mg/kg) and xylazine (10 mg/kg), given atropine (0.05 mg/kg) to help with respiration, and placed on a heating pad. A post-auricular incision was made, the temporal bone was exposed by blunt dissection, and the bulla was opened. After confirmation of a normal uninfected ear, the bulla was temporarily closed with a small cotton ball and the recording and percutaneous connector anchoring screws were placed on the skull. A midline incision was made on the skull, and the skin and pericranium were retracted for view of bregma. Screws were placed at three points around the bregma and these were used to secure a small inverted bolt, which could serve as a ground and/or anchor for the electrode base. This bolt was secured permanently with methyl methacrylate. Additional screws were placed at the midline, 1 cm caudal to bregma, and one 1 cm from the midline and slightly behind bregma on the implant side.

Once all hardware was secured, the cotton ball sealing the bulla was removed and a portion of the lateral wall of the basal turn of the scala tympani of the cochlea was removed with a small diamond bur until the basilar membrane and first turn could be seen clearly and there was room for insertion of all beams of the PIAT. Perilymph was wicked out with a cotton pledget for viewing of the cochlear structures and a dummy implant was used to gage opening size and angle before insertion of a functional implant. Once the opening was large enough, the percutaneous connector was temporarily affixed to the skull surface and anchor screws with Durelon cement. Then, the implant was gripped with forceps and inserted into the scala tympani to the depth of the first turn following the lateral edge of the scala tympani and angled such that the beams were parallel with the basilar membrane surface. Figures 3C, 4D showed the location where the PIAT was implanted. A small muscle plug was placed on top of the implant and into the cochleostomy opening in an attempt to seal the opening and prevent leakage of perilymph. With Durelon cement, the implant was secured to the bulla and the bulla opening sealed. For grounding purposes, a flamed 5 T platinum iridium ground ball electrode was tucked into the neck muscle overlying the temporal bone. The skin incision was closed over the implant and ground wires and the implant was tested for functionality, as detailed below. Once functionality was confirmed, the percutaneous connector was permanently secured to the skull and the anchor screws with methyl methacrylate and the animal was moved to a sound-attenuating booth for the remainder of the testing. Supplemental doses of the ketamine, xylazine and atropine anesthesia regime listed above were given throughout testing to maintain a consistent depth of anesthesia to perform the electrophysiology and implant recordings.

### Hearing Condition of the Tested Animal

As discussed in the Results, the PIAT was not affected by the CM. Therefore, the device can work in both deafened and non-deafened animals. In the test presented in this paper, the guinea pig was not deafened. However, the hearing threshold was elevated significantly. To evaluate the hearing condition of the ear after PIAT implantation, Auditory Brainstem Responses (ABRs) were recorded and the hearing threshold for multiple frequencies determined. The thresholds for the frequencies tested were 100 dB (2 kHz), 80 dB (4 kHz), 70 dB (8 kHz), 60 dB (10 kHz), 85 dB (12 kHz), and 75 dB (16 kHz) SPL. These were considerably elevated compared to normal thresholds. These results showed the hearing was impaired, and that the CM should be fairly small; smaller than 1 mV as has been measured in healthy animals^48^. During the ABR test, the animal was kept anesthetized and placed on a heating blanket in a sound attenuating booth. Needle electrodes were positioned subcutaneously at the vertex and bilaterally underneath the pinna. An acoustic transducer with a speculum was placed just inside the tragus and pointed toward the tympanic membrane. Tone bursts were presented and responses were recorded using a Tucker Davis Technology System 3 BioSig32 system.

### *In vivo* Measurement of Voltage Output of the PIAT inside the Cochlea of a Live Guinea Pig

Figures 3B, 4D illustrate the location where the PIAT was implanted. After implantation, a speaker (ES 1, Tucker-Davis Technologies, USA) was directed at the tympanic membrane via a speculum to the ear canal and a series of frequencies ranging from 1–14 kHz at levels from 80–95 dB SPL were played. While playing these acoustic excitations, a lock-in amplifier (SR830, Stanford Research System, USA) was used to measure the voltage output of the PIAT located inside the cochlea. As in the benchtop testing, the custom-made amplifier was used to buffer and amplify the PIAT voltage response. This amplification gain was again factored out. *In vivo* noise was obtained by playing sound in air, with the speaker pointing away from animal and the ear canal occluded. The electrical impedance of the PIAT was measured with an LCR meter (E4980A, Agilent, USA) before the implantation and every 30 minutes during the *in vivo* experiment to continuously monitor the condition of the device.

### *Post Mortem* Cochlear Dissection and *Ex Vivo* Actuation in Air

At the completion of testing, the animal was euthanized and stored in a freezer. Two days later, the implanted cochlea was dissected to assess implant insertion location and the status of each of the implant’s beams (Fig. 3C,D). The cochlea was approached by removing the ear canal and the outer wall of the otic capsule. The cochlea was then scored on the surface with a scalpel blade and starting at the apex the outer boney wall was gently removed with a pair of fine forceps exposing the coils of the cochlear spiral, basilar membrane, and modiolus. Each cochlear turn was inspected for damage caused by the implant and the angle and general location of the implant. Once the basal most turn was exposed, the status of each beam was assessed. Figure 3D shows the assessment and status of each beam. Finally, the modiolus was removed for complete visualization of the implant in the basal turn and for gentle removal of the implant for functionality testing post-removal. The PIAT probe was actuated by voltage and tip deflection of the beams was measured by an LDV.

## Supplementary information

Figure S1
