# Supplementary material for: Voltage readout from a piezoelectric intracochlear acoustic transducer implanted in a living guinea pig
Source: Sci Rep. 2019 Mar 6;9:3711. doi: 10.1038/s41598-019-39303-1 (PMC6403321; doi:10.1038/s41598-019-39303-1)
Supplement: Supplementary file 1 — Figure S1 [file 41598_2019_39303_MOESM1_ESM.pdf]

Supplementary Materials

Voltage readout from a piezoelectric intracochlear acoustic transducer implanted in a living guinea pig

Chuming Zhao[1], Katherine E. Knisely[1], Deborah J. Colesa[2], Bryan E. Pfingst[2], Yehoash Raphael[2], and Karl Grosh[1,3*]

[1]Department of Mechanical Engineering, University of Michigan, Ann Arbor, MI, 48109, USA
[2]Kresge Hearing Research Institute, Department of Otolaryngology - Head and Neck Surgery, University of Michigan, Ann Arbor, MI, 48109, USA
[3]Department of Biomedical Engineering, University of Michigan, Ann Arbor, MI, 48109, USA
[*]grosh@umich.edu

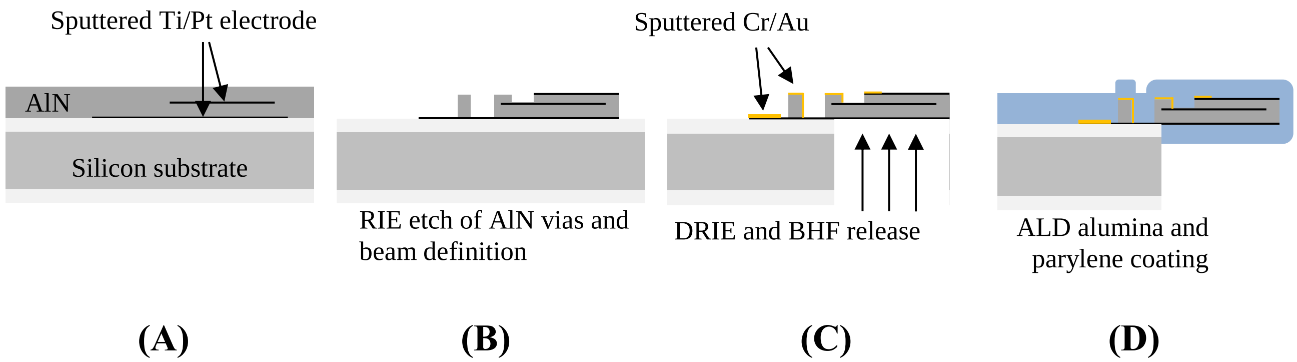

Fig. S1. MEMS fabrication process. (A) AlN deposition and Ti/Pt electrode layer deposition and patterning. (B) RIE etch of the AlN layers to define beam dimensions and create vias to the middle and bottom electrode layers. The top AlN layer was overetched as described in the Methods. (C) Cr/Au deposition for circuit layout connection, followed by DRIE and BHF release. (D) Waterproof coating that consists of ALD alumina and parylene conformal coating after device release.
